# Microscopic Observation of Membrane Fusion between Giant Liposomes and Baculovirus Budded Viruses Activated by the Release of a Caged Proton

**DOI:** 10.3390/membranes13050507

**Published:** 2023-05-11

**Authors:** Misako Nishigami, Yuki Uno, Kanta Tsumoto

**Affiliations:** Division of Chemistry for Materials, Graduate School of Engineering, Mie University, 1577 Kurimamachiya-cho, Tsu 514-8507, Mie, Japan; misakonishigami2022@gmail.com (M.N.);

**Keywords:** giant vesicle, baculovirus, membrane fusion, confocal laser scanning microscopy, fluorescence microscopy, caged proton

## Abstract

Baculovirus (*Autographa californica* multiple nucleopolyhedrovirus, AcMNPV) is an envelope virus possessing a fusogenic protein, GP64, which can be activated under weak acidic conditions close to those in endosomes. When the budded viruses (BVs) are bathed at pH 4.0 to 5.5, they can bind to liposome membranes with acidic phospholipids, and this results in membrane fusion. In the present study, using the caged-proton reagent 1-(2-nitrophenyl)ethyl sulfate, sodium salt (NPE-caged-proton), which can be uncaged by irradiation with ultraviolet light, we triggered the activation of GP64 by lowering the pH and observed membrane fusion on giant liposomes (giant unilamellar vesicles, GUVs) by visualizing the lateral diffusion of fluorescence emitted from a lipophilic fluorochrome (octadecyl rhodamine B chloride, R18) that stained viral envelopes of BVs. In this fusion, entrapped calcein did not leak from the target GUVs. The behavior of BVs prior to the triggering of membrane fusion by the uncaging reaction was closely monitored. BVs appeared to accumulate around a GUV with DOPS, implying that BVs preferred phosphatidylserine. The monitoring of viral fusion triggered by the uncaging reaction could be a valuable tool for revealing the delicate behavior of viruses affected by various chemical and biochemical environments.

## 1. Introduction

For decades, it has been reported that novel viruses infectious to human beings occasionally emerge, and many of them seem to belong to a family of enveloped viruses [1], which include influenza virus, coronavirus, etc. In general, the pathway of viral infection is strictly dependent on the kind of virus; however, any viral infection with enveloped viruses should basically involve membrane fusion between enveloped virions and the target membranes of host cells. In infection, both cellular and endosomal membranes can be targeted by virions through the interaction of specific receptor molecules expressed on target host membranes and fusogenic proteins on viral envelopes [2,3,4]. Fusogenic proteins bind to the target membranes in an activated form and make both the lipid bilayers juxtaposed and perturbed to cause membrane fusion, which can be followed by transfer of viral nucleic acids condensed as genomic materials from virions into the host cellular cytoplasm. Membrane fusion is an essential step toward viral entry, and thus many strategies have been studied to inhibit this step to block the propagation of infectious viruses with envelopes [5].

Nucleopolyhedrovirus is an insect-enveloped virus that is a type of baculovirus, and it is widely used in biotechnology for the production of recombinant proteins [6]. *Autographa californica* multiple nucleopolyhedrovirus (AcMNPV) is often used for this purpose. Therein, a gene coding the protein polyhedrin is removed and a foreign gene is recombinantly introduced into its locus [6,7,8,9]. The polyhedrin is highly expressed in nature to protect occlusion-derived viruses (ODVs) in the exterior environment when the genomes encapsulated inside ODV are transferred from larva to larva. The recombinant gene can be controlled under the strong promotor of the polyhedrin to produce substantial target proteins with infected insect culture cells such as Sf9 cells. Regarding the recombinant baculovirus, it can no longer produce ODVs because the gene of the polyhedrin *Ac-PH* is replaced by a target gene. In addition to ODVs, baculovirus can form budded viruses (BVs) in its life cycle [6,9]. BVs are needed for cell-to-cell infection inside larvae and bud from cells to obtain their envelopes originating from the cell membranes of the host-infected insect cells. Since BVs can be easily produced using culture cells, they have been used as bionanomaterials [10,11] for tracking intracellular trafficking [12] and transferring recombinant membrane proteins to artificial lipid membranes [13,14,15,16,17]. 

Infection of culture cells by BVs is the first step in the preparation of BV particles, and therefore it is important to analyze biochemical and biophysical tendencies and preferences in BV-cell membrane and/or -lipid membrane fusion, which is induced by the fusogenic envelope glycoprotein GP64 expressed on baculovirus budded virions [18,19]. Activated when the pH is lowered to ~4.0–5.5, GP64 expressed on BVs bound to membranes can acquire fusogenic ability, and then fuse with such target membranes, when the membranes contain an acidic phospholipid such as phosphatidylglycerol or phosphatidylserine, the latter of which is considered to serve as a receptor for BVs [18,20].

The aforesaid properties are well-known since membrane fusion has been investigated using cells and artificial vesicles [13,14,15,16] and supported lipid membranes [17] with regard to the stationary case before and after changing pH conditions, but membrane fusion has not been monitored after BV activation by lowering the pH. In the present study, using a caged-proton reagent (1-(2-nitrophenyl)ethyl sulfate, sodium salt; NPE-caged-proton), which can be uncaged by irradiation with ultraviolet light [21], we set the time at which GP64 started to be activated. We could observe membrane fusion on giant liposomes (giant unilamellar vesicles, GUVs) by visualizing the lateral diffusion of fluorescence emitted from a lipophilic fluorochrome (octadecyl rhodamine B chloride; R18) that stained the viral envelopes of BVs. Interestingly, the behavior of BVs prior to fusion varied depending on the content of GUVs, which implies the existence of a preference regarding fusion to some phospholipid species.

## 2. Materials and Methods

### 2.1. Materials

1,2-Dioleolyl-*sn*-glycero-3-phosphocholine (DOPC), 1,2-dioleoyl-*sn*-glycero-3-phospho-L-serine, sodium salt (DOPS-Na), and 1,2-dioleoyl-*sn*-glycero-3-phospho-(1′-*rac*-glycerol) sodium salt (DOPG) were purchased from NOF Corporation (Tokyo, Japan; DOPC and DOPS) and Avanti Polar Lipids (Alabaster, AL, USA), respectively, for the preparation of lipid membrane vesicles (giant liposomes, GUVs). The lipid was dissolved in chloroform (Spectrosol^®^, FUJIFILM Wako Pure Chemical Corporation, Osaka, Japan) and stored in a deep freezer at around −80 °C prior to use in the following experiment. Calcein and octadecyl rhodamine B chloride (R18), which were purchased from Thermo Fisher Scientific (Waltham, MA, USA), were used to fluorescently label the interior of GUVs and the envelopes of baculovirus budded virions, respectively. 1-(2-Nitrophenyl)ethyl sulfate, sodium salt (NPE-caged-proton; Bio-Techne, Minneapolis, MN, USA) was used for the caged-proton compound, which was activated by ultraviolet-light (UV) irradiation to induce the release of the proton, leading to a steep decrease in pH.

### 2.2. Preparation of Baculovirus Budded Viruses

The budded viruses (BVs) of the baculovirus (AcMNPV) were prepared according to our previous reports [10,11]. Sf9 cells originated from *Spodoptera frugiperda* were purchased as host cells from Thermo Fischer Scientific (Waltham, MA, USA). For BV amplification, the cells were cultured at 27 °C in two 25 cm^2^ plastic flasks (Sumitomo Bakelite, Co., Ltd., Tokyo, Japan), each of which contained 5 mL of Sf-900 III serum-free medium (SFM, Thermo Fischer Scientific) to almost full confluence. Scraped from the bottom, the cell suspension was added to a plastic vent-cap flask with baffles (Corning Inc., Corning, NY, USA), which contained 100 mL Sf-900 III SFM, and further cultured for 6–8 days in a shaking incubator (27 °C, 150 rpm; Taitec Corporation, Saitama, Japan) prior to the following infection. A portion (1 mL) of the supernatant of the seed BVs of wild-type AcMNPV was added to the above flask, and the cells were incubated for an additional 4–5 days. The liquid culture was subjected to centrifugation (2000× *g*, 5 min), and the supernatant was ultracentrifuged using a Beckman Coulter L-70 ultracentrifuge (107,000× *g*, 30 min, 15 °C; Beckman Coulter Inc., Brea, CA, USA) to precipitate amplified BVs. The resulting pellet was resuspended with an aliquot of 1 × PBS (phosphate-buffered saline; 1 mM Na_2_HPO_4_, 10.5 mM KH_2_PO_4_, 140 mM NaCl, 40 mM KCl (adjusted with NaOH aq. to pH 6.2)). The suspension was placed on a step-wise sucrose density gradient (10 wt% (6.3 mL), 15 wt% (6.3 mL), and 20 wt% (18.9 mL) in 1 × PBS) and subjected to ultracentrifugation (38,400× *g*, 30 min, 15 °C). Since BV particles accumulated in the zones containing 10% and 15% sucrose, these two fractions, upper and lower, were recovered separately, and diluted with 1 × PBS and subjected again to ultracentrifugation (107,000× *g*, 30 min, 15 °C). The resulting precipitates were resuspended in 100–300 µL of 1 × PBS.

### 2.3. Fluorescent Labeling of BV Particles

The BV particles were stained with the lipophilic fluorochrome R18 according to the protocol reported previously [13]. A portion of 4 mM R18 in ethanol was added to the BV suspension from the lower fraction at a ratio of 40 nmol of R18 to 1 mg of the BVs, which was determined by a Bio-Rad Protein Assay Kit (Bio-Rad Laboratories, Inc., Hercules, CA, USA) using bovine serum albumin (BSA) as a standard. The suspension was kept inverted repeatedly for 1 h at room temperature. BVs mixed with R18 were loaded onto the column (1.0 cm × 30 cm) with Sephadex^TM^ G-50 (Cytiva, Tokyo, Japan) equilibrated by 1 mM phosphate buffer (Na_2_HPO_4_/NaH_2_PO_4_, pH 7.2)/150 mM NaCl, and eluted and collected with the same buffer solution at 1 mL/min in a cool room (around 5 °C). Fractions containing R18-labeled BVs were recovered based on their fluorescence intensity (Ex 560 nm; Em 590 nm) observed using a fluorescence spectrophotometer (F-2500, Hitachi, Tokyo, Japan).

### 2.4. Preparation of GUVs Using the Droplet-Transfer Method

**Figure 1:**
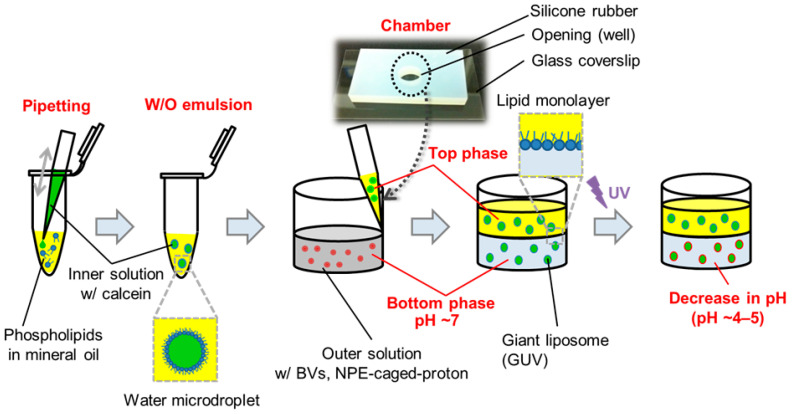
Schematic illustration of the experimental procedure for preparing GUVs that could be observed using a fluorescence confocal laser scanning microscope. Initially, the solution of the bottom phase, which would, in turn, be an outer solution, contained both BVs and NPE-caged-protons. After the preparation of GUVs by the transfer of aqueous droplets through an oil/water interface, the chamber was irradiated by UV light leading to the release of protons from the caged molecules.

GUVs were prepared using the droplet-transfer method [16], which was previously reported by pioneering research groups [22,23]. We also used it to investigate membrane fusion between phospholipid membranes and BVs. Briefly, a 100 µL portion of 1 mM DOPC and/or 1 DOPS or 1 mM DOPG in chloroform was evaporated to form dry films in a glass microtube under flow of N_2_ gas. The dry films were bathed with 200 µL of mineral oil and sonicated for 1 h at 50 °C. The experimental procedure is illustrated in Figure 1. First, the suspension of phospholipid in mineral oil was transferred to a microtube, and a small portion (5 µL) containing 10 µM calcein in the 1 mM phosphate buffer (pH 7.2)/150 mM NaCl was added for a solution entrapped within inner phases of GUVs. By vigorous pipetting using a micropipette, the aqueous solution was emulsified into small water-in-oil (W/O) droplets, a portion (30 µL) of which was gently placed as a top phase on the following bottom phase (30 µL) in a chamber consisting of a glass cover slip (30 mm × 40 mm, Matsunami Glass Ind., Ltd., Kishiwada, Japan) and a piece (20 mm × 30 mm; 5 mm in thickness) of silicone rubber with an opening (6 mm in diameter). The solution for the bottom phase was prepared by mixing 20 µL of NPE-caged-proton (0–3 mM final concentration) in the 1 mM phosphate buffer (pH 7.2)/150 mM NaCl and 80 µL of the suspension of R18-labeled BVs. In general, phospholipid molecules suspended in mineral oil could spontaneously assemble to form lipid monolayers on aqueous microdroplets. The monolayer-wrapped W/O droplets in the top phase moved to the interface on the bottom phase, where another lipid monolayer could also form; thus, as the W/O microdroplet transferred through the interface, these lipid monolayers met each other to generate a GUV beneath the interface.

### 2.5. Microscopic Observation of GUVs Mixed with Fluorescenty Labeled BVs

An inverted confocal laser scanning microscope (CLSM; Carl Zeiss LSM710; Carl Zeiss AG, Oberkochen, Baden-Württemberg, Germany) equipped with an objective lens (Plan-Apochromat, 10×) was used to observe calcein-containing GUVs and R18-labeled BVs in time-lapse mode. Peaks at 488 nm and 561 nm were selected for excitation of calcein (Em 493–556 nm) and R18 (Em 566–680 nm), respectively. The working distance of the objective lens is long enough to observe the position close to the interface, which is relatively far from the bottom cover slip. For this purpose, the objective lens of low magnification was required to be selected in the present situation. To uncage the NPE-caged-proton, the opening of the chamber was directly irradiated by UV using an LED pen-type light (365–375 nm; STYLUS^®^ UV, Streamlight, Inc., Eagleville, PA, USA) from 2 min or 5 min to 14.5 min after the observation was started. The microscopic images were acquired and analyzed using ZEN (Carl Zeiss AG) and ImageJ (Rasband, W.S., ImageJ, National Institutes of Health, Bethesda, MD, USA, https://imagej.nih.gov/ij/ (accessed on 1 May 2023), 1997–2018). As shown in the next section, we evaluated how proton concentrations, or pH values, changed with time under UV irradiation of the phosphate-buffered solution containing the caged proton reagent at various concentrations (0–3 mM).

## 3. Results and Discussion

### 3.1. Uncaging Proton in the Outer Phase Solution under UV Irradiation

**Figure 2:**
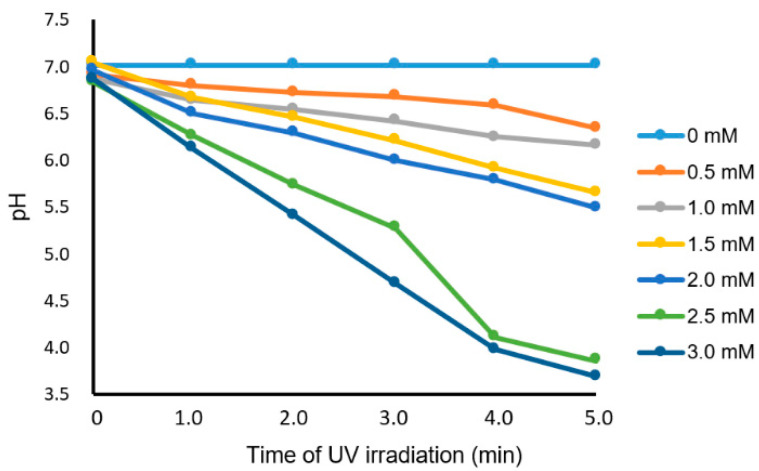
Time-course of pH vs. the duration of UV irradiation in the aqueous solution containing NPE-caged protons at the indicated concentrations.

The fusogenic activity of GP64 expressed on BV envelopes was activated at low pH conditions when membrane fusion occurred between the BVs and artificial lipid membranes of liposomes [18]. Therefore, we verified whether UV irradiation of the solution containing caged-proton triggered a decrease in pH to an appropriate value. A small portion (30 µL) of the NPE-caged-proton dissolved at various concentrations in the 1 mM phosphate buffer (pH 7.2)/150 mM NaCl was placed on the detector of the pH meter (Pocket-sized pH meter S2K922; Isfetcom Co., Ltd., Saitama, Japan) under UV irradiation with the previously mentioned light source. Figure 2 shows the time-course curves of the pH of solutions containing the indicated concentrations of the NPE-caged-proton during the uncaging reaction. As reported previously, the fusogenic function of GP64 is expressed at around pH = 5.5 and below [13,14]. Though this apparatus for UV irradiation was simple, the buffer solution could become acidic enough to activate GP64 on the microscopic stage within 5 min, when a concentration of 2 mM or more was selected. 

### 3.2. Time-Lapse Observation of Membrane Fusion of a GUV with BVs That Could Be Activated by a Photocontrollable Decrease in pH

**Figure 3:**
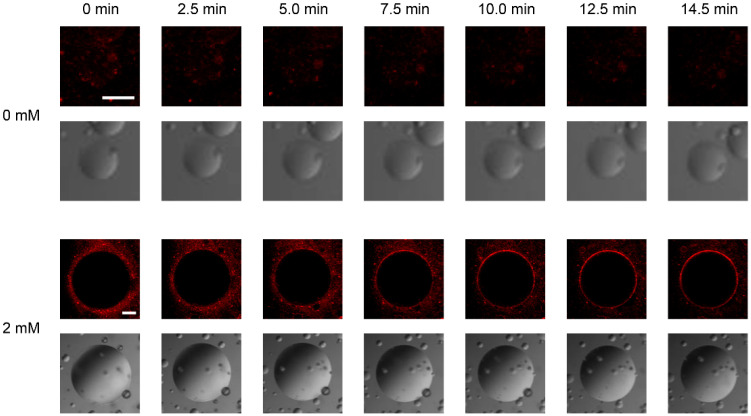
Typical CLSM images of GUVs (DOPC/DOPS = 1:1) mixed with BVs that have been fluorescently labeled with the fluorochrome R18. The indicated concentrations, 0 mM (none) and 2 mM, refer to the concentration of NPE-caged-protons added to the outer solution of the GUVs. The time shows the elapsed time after the observation started. The sample began to be illuminated by UV light at 5.0 min. Each panel consists of R18 fluorescent images (upper panels) and differential interference contrast (DIC) images (lower panels) of the same GUVs. Bar is 50 µm.

The pH of the buffered solution could be controlled by uncaging the NPE-caged-proton under UV irradiation, and thus we next tried to monitor membrane fusion of individual GUVs with BVs that was triggered through the activation of GP64 proteins. Figure 3 clearly demonstrates that the uncaging reaction can trigger membrane fusion of BVs to GUVs under CLSM observation. Without the NPE-caged-proton, UV irradiation did not affect BV behavior, as reflected by the dispersion of small fluorescent dots around GUVs during the observation. In contrast, in the presence of the caged proton (2 mM), UV irradiation started, and the contour line of the target GUV became more obvious; interestingly, the typical image of a target GUV observed by CLSM suggests that fluorescence may have spread from a point where R18-stained BVs fused with the lipid membrane of a GUV toward their opposite pole. The contour circumference of the GUV emitted smooth fluorescence after 5.0–7.5 min. R18 exhibits a self-quenching behavior when it is highly accumulated on lipid membranes. Here, BV envelopes were stained with R18 at so relatively high concentration that the fluorescence was partially quenched. Once the BVs fused with GUV membranes, the fluorescence intensity increased, because the dye molecules laterally diffused over the GUVs, and their density decreased.

**Figure 4:**
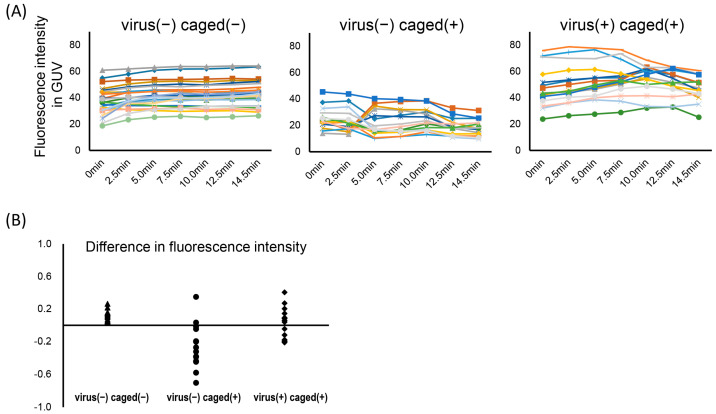
Changes in fluorescence intensity of calcein encapsulated inside GUVs in the presence (+) or absence (−) of BVs and NPE-caged-protons. (**A**) The fluorescence intensity of calcein was monitored after the start of UV irradiation. Each line represents the time-course data of a single GUV. (**B**) The difference in relative fluorescence intensity with each GUV was plotted under the indicated conditions. The difference is calculated by dividing the intensity at 14.5 min minus that at 0 min by that at 0 min. Typical fluorescence images of GUVs entrapping calcein are provided in Figure S1 in the Supplementary Material. The lines and dots are corresponding to individual GUVs.

UV irradiation did not affect the quality of GUVs in the present experiment (Figure 3), whereas the increase in proton concentration under UV irradiation damaged GUV membranes. Figure 4 shows the time-course profile of the florescence intensity of calcein entrapped inside GUVs under UV irradiation. Without BV or the caged proton, calcein remained entrapped for several minutes. In contrast, when the caged proton was uncaged in the absence of BVs, the released proton should induce permeation or leakage of calcein, since the fluorescence of calcein tended to decrease. Interestingly, this trend was attenuated when the caged proton was included with BVs, indicating that membrane fusion with BVs might act as buffering materials when the lipid membranes suffer from acidic damage that caused partial leakage of calcein. This implied that the membrane fusion observed here should be induced by a native function of GP64, which can moderately cause fusion with GUVs with less leakage [16]. We monitored a selected GUV in a single field of view and, as implied in Figure 4, GUV images were observed with dispersion, and so a trend can be discussed.

### 3.3. Preference of Phosholipids in Membrne Fusion betweem BVs and GUVs

As the affinity of BVs for actual host cells is a significant factor that determines the infection behavior, the preferences of phospholipid of BVs and how BVs are associated with lipid membranes of host cells are intriguing problems. Some experiments on membrane fusion with liposomes have suggested that BVs can be associated with phosphatidylserine, which is considered to be a natural receptor molecule [18,20], more efficiently than phosphatidylglycerol, which is an acidic phospholipid that can serve as a receptor molecule [14]. In the previous work, GUVs were mixed with fluorescently labeled BVs, incubated for a while, and then subjected to microscopic observation; thus, it was not possible to see a state of BVs just prior to membrane fusion. On the other hand, since we adopted the uncaging reaction to trigger the activation of GP64, we could see the state prior to membrane fusion. 

**Figure 5:**
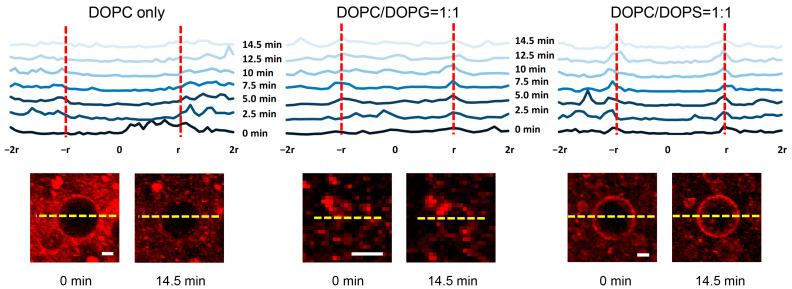
Typical CLSM images of GUVs (DOPC only, DOPC/DOPG = 1:1, DOPC/DOPS = 1:1 molar ratio) mixed with BVs that were fluorescently labeled with the fluorochrome R18 at 2 mM NPE-caged-proton. The solutions were irradiated by UV for the indicated time. Each panel consists of the top profiles, which show the time-lapse change in R18 fluorescence along the dashed line (yellow) crossing the GUV (the intersections are indicated by red dashed lines in the upper panel), and the bottom images, which show the corresponding GUVs at 0 min and 14.5 min. In the case of DOPC/DOPG, the GUV that was smaller compared to the others was observed using the objective lens with low magnification, and the image looks more pixelated. Bar is 20 µm.

Figure 5 shows typical CLSM images of GUVs containing different acidic lipids to which BVs would be bound and fused. After UV irradiation, BVs with activated GP64 underwent membrane fusion, which was evident judging from an increase in fluorescence intensity, or growing peaks, on both of the lipid bilayers of a GUV containing DOPG and DOPS, while such an increase was not observed with a GUV of DOPC. Since DOPC is electrostatically neutral, BVs could show slight affinity for the lipid membrane prior to triggering, but the association was not enhanced even at 14.5 min. In contrast, GUVs containing acidic phospholipids such as DOPG and DOPS possess negative charges, which may cause a repulsive interaction between GUVs and BVs that was also negatively charged at a neutral pH [24]; nevertheless, BVs appeared to accumulate near the membranes of GUVs with DOPS, i.e., just around the outer side of the GUV surface, prior to the uncaging reaction, which could imply a native preference of BVs for DOPS.

## 4. Conclusions

We have demonstrated that membrane fusion between BVs and GUVs could be triggered using the uncaged reaction of a caged proton. Membrane fusion of a single GUV with R18-stained BVs was monitored just after UV irradiation, and the fluorescence could spread over the contour circumference smoothly. Direct observation of calcein entrapped inside GUVs before and after triggering fusion implied that membrane fusion with BVs attenuated the damage to GUVs exposed to a decrease in pH. Under close monitoring of the behavior of BVs prior to the triggering of membrane fusion by uncaging, BVs appeared to be accumulated around a GUV containing DOPS compared to that with only DOPC or DOPC plus DOPG, which implies that BVs exhibited a preference for phosphatidylserine, similar to BVs in nature. However, we here set the composition of phospholipid to facilitate microscopic monitoring the membrane fusion triggered by the uncaging reaction according to the similar composition reported previously in our work of BV–GUV membrane fusion [14]. It should also be noted that the phospholipid content adopted here is different from that observed in nature [25,26]; especially, the composition of phosphatidylserine is larger than that in natural situations, and their distribution is supposed to be homogeneous between inner and outer leaflets of lipid membranes of GUVs, which trend is different from the preference that phosphatidylserine molecules are made to be more partitioned to the inner leaflets of living cell membranes with enzymes that can maintain the asymmetric distribution of phospholipid [27]. Since the present experiment was conducted for an artificial model for viral membrane fusion qualitatively, the further investigation and improvement should be required for qualitative analysis through employing comparative studies with living host cells. The monitoring of viral fusion triggered by the uncaging reaction could serve as an assay tool to reveal the detailed behavior of viruses affected by various chemical and biochemical environments. In the present study, we only adopted baculovirus BV-enveloped virions, and unfortunately, we do not have any evidence for whether this monitoring experiment protocol could be validated. It should be noted that plain GUVs used here are too simple to develop any sophisticated intracellular membrane traffic systems such as endocytosis, which usually serve as a main pathway for viral infection. Hence, further experiments are needed for extending the range of species of enveloped viruses to which the method is applicable. 

## Data Availability

Not applicable.

## Supplementary Materials

The following supporting information can be downloaded at: https://www.mdpi.com/article/10.3390/membranes13050507/s1, Figure S1: Time-course CLSM images of calcein-containing GUVs.
