# Supplementary material for: Microscopic Observation of Membrane Fusion between Giant Liposomes and Baculovirus Budded Viruses Activated by the Release of a Caged Proton
Source: Membranes (Basel). 2023 May 11;13(5):507. doi: 10.3390/membranes13050507 (PMC10220649; doi:10.3390/membranes13050507)
Supplement: Supplementary file 1 [file membranes-13-00507-s001.zip › membranes-1955745-supplementary.pdf]

Supplementary material

Microscopic observation of membrane fusion between giant liposomes and baculovirus budded viruses activated by the release of a caged proton

Misako Nishigami [1], Yuki Uno [1] and Kanta Tsumoto [1,*]

1 Division of Chemistry for Materials, Graduate School of Engineering, Mie University, 1577 Kurimamachiya-cho, Tsu, Mie 514-8507, Japan

* Correspondence: tsumoto@chem.mie-u.ac.jp; Tel.: (+81 59 231 9429)

Time-course CLSM images of calcein-containing GUVs are provided in the following figure (Figure S1). Calcein entrapped in GUVs emitted green fluorescence. Though large dispersion and morphological changes observed in GUVs could make it difficult to predict meaningful behavior, it seems that the fluorescence of calcein entrapped inside GUVs might be constant without viruses or caged proton, whereas it decayed with uncaged proton for 14.5 min as depicted in Figure 4A of the main text. Over the former half of the period, the decay might be attenuated with BVs as mentioned in the main text. Figure S1 consists of three panels: The CLSM images of GUVs without baculovirus BVs or caged proton (A); without baculovirus BVs and with caged proton (B); and with baculovirus BVs and caged proton (C). CLSM images were acquired at the indicated time after the uncaged reaction by UV irradiation.

(A) virus (-) caged (-)

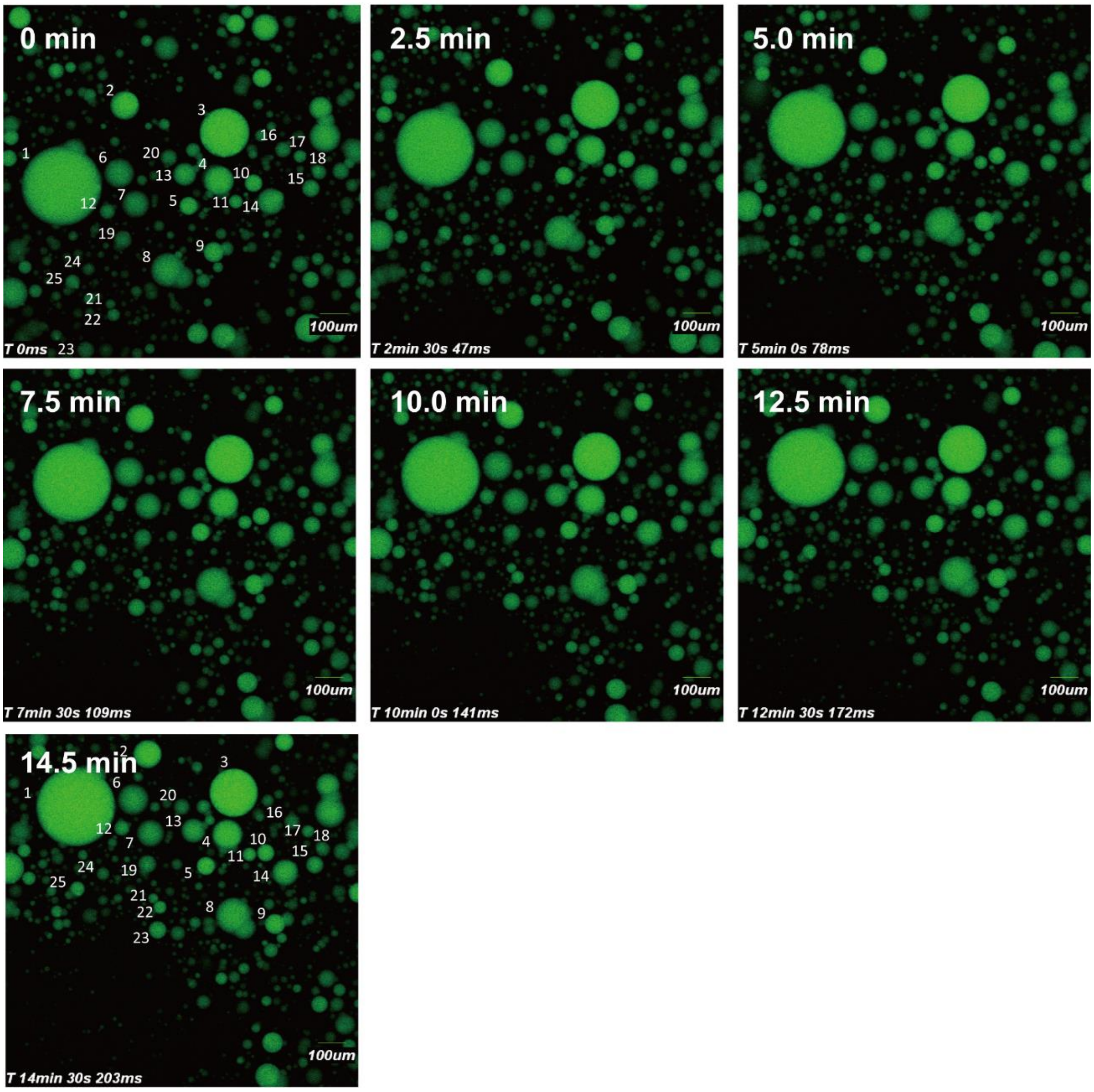

Figure S1A. CLSM images of GUVs without viruses or caged proton acquired at the indicated time after the uncaged reaction by UV irradiation. The number indicated in the image at 0 and 14.5 min means the numbered vesicle was monitored in Figure 4 of the main text.

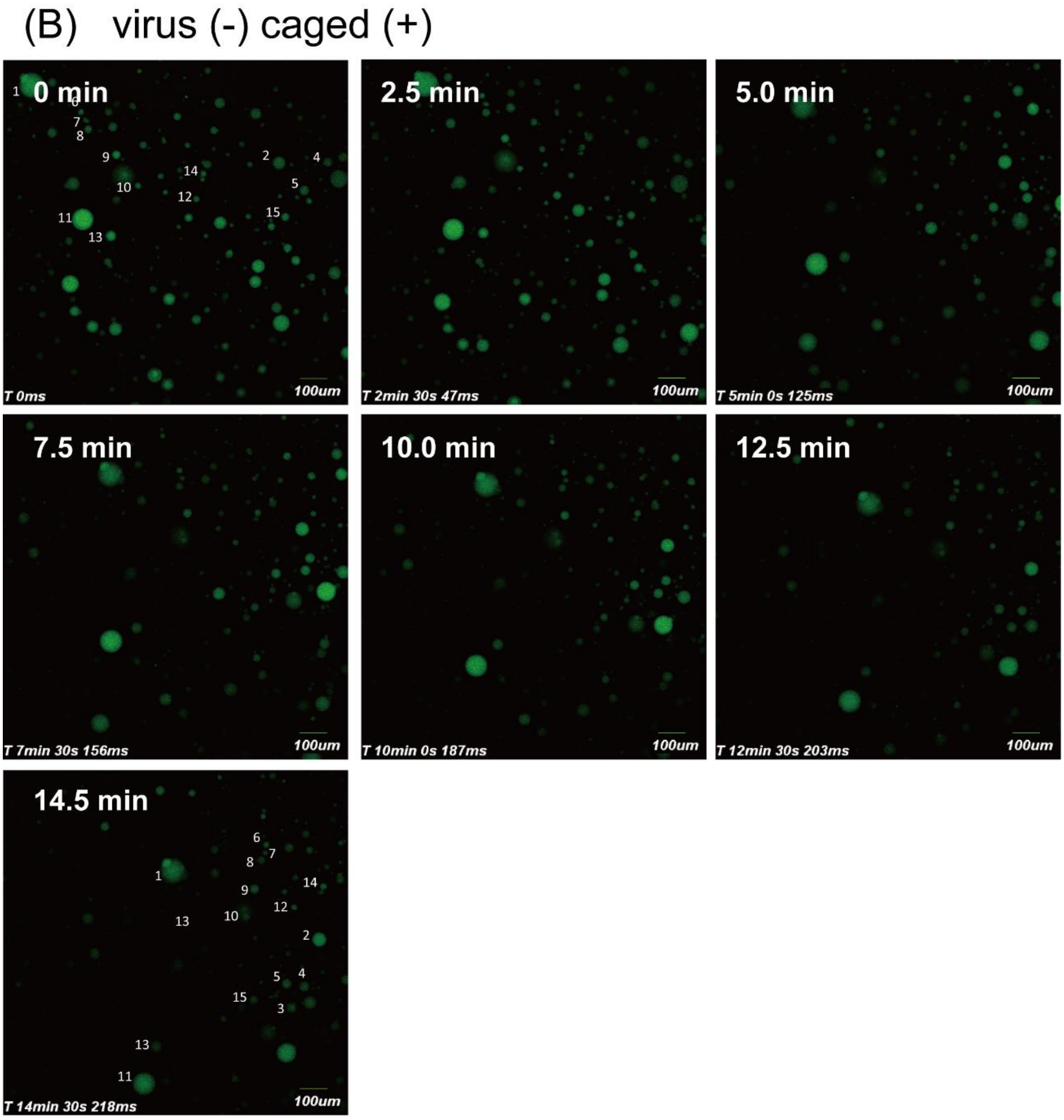

Figure S1B. CLSM images of GUVs without viruses and with caged proton acquired at the indicated time after the uncaged reaction by UV irradiation. The number indicated in the image at 0 and 14.5 min means the numbered vesicle was monitored in Figure 4 of the main text.

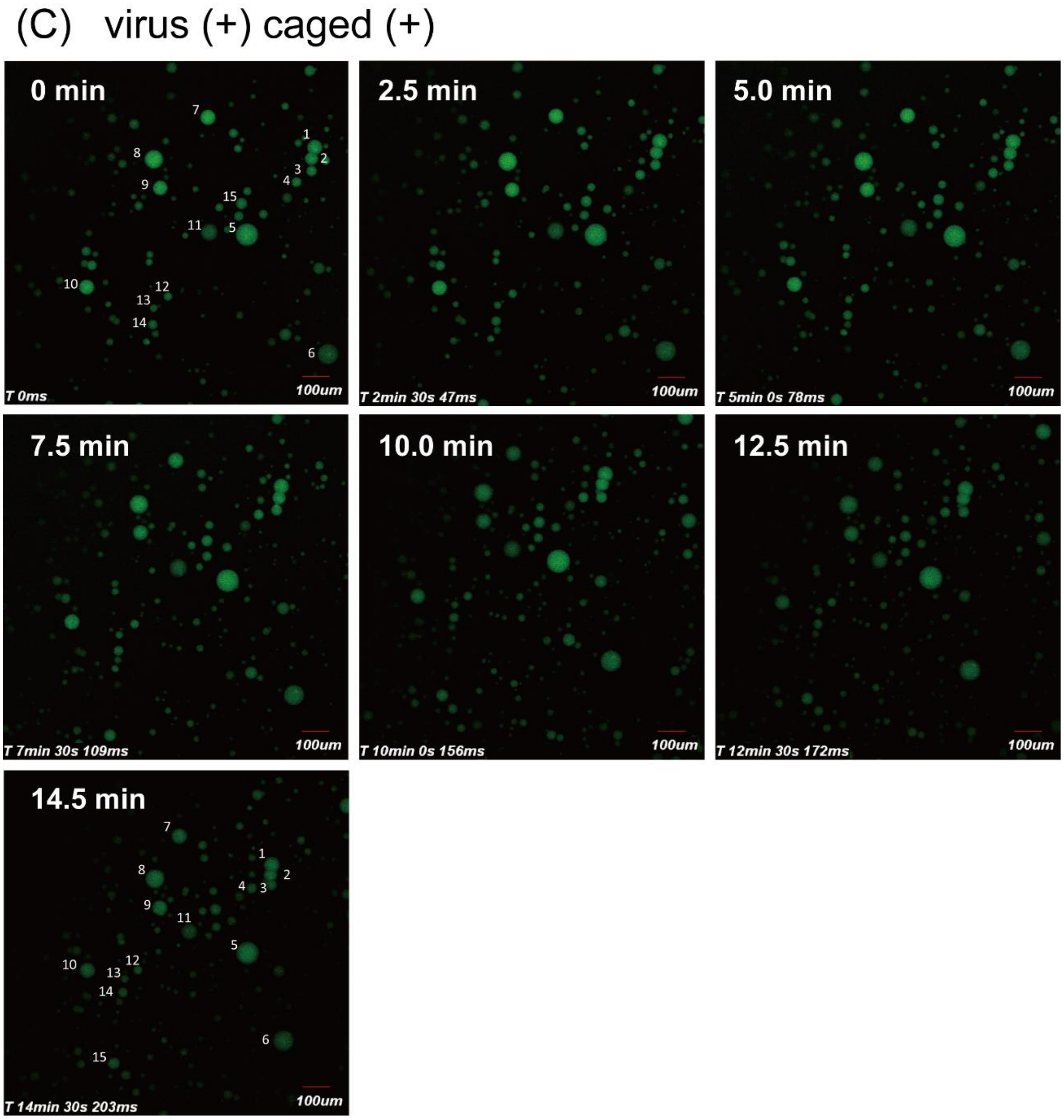

Figure S1C. CLSM images of GUVs with viruses and caged proton acquired at the indicated time after the uncaged reaction by UV irradiation. The number indicated in the image at 0 and 14.5 min means the numbered vesicle was monitored in Figure 4 of the main text.
